# Enhancing Alzheimer’s Disease and Related Dementia Burden Projections and Policy Targeting in China

**DOI:** 10.34133/hds.0436

**Published:** 2026-04-13

**Authors:** Chuanqian Liu

**Affiliations:** Department of Neurosurgery, Xiongan Xuanwu Hospital, Xiongan New Area 070001, China.

I highly appreciate Liu et al.’s impactful study on projecting Alzheimer’s disease and related dementias’ (ADRDs’) prevalence and economic burden across Chinese provinces [1]. Its integration of national longitudinal survey data with provincial statistics and distinction between formal and informal care costs fills a critical gap for region-specific policy formulation. To enhance reproducibility, I suggest clarifying standardized cutoffs for cognitive impairment across education strata in the China Health and Retirement Longitudinal Study (CHARLS) in future analyses.

Liu et al.’s findings highlight the urgent need for targeted interventions, and I propose 3 focused extensions to boost practical value. First, integrating modifiable risk factors such as physical activity, diet, and air pollution [2,3] could refine prevalence projections. These factors are well-documented to influence ADRD risk but were not incorporated into the study’s projection model due to data limitations. Practically, lifestyle data can be supplemented by harmonizing intermittent CHARLS lifestyle records, while air pollution data are publicly available from provincial environmental monitoring departments, making this extension feasible. Second, developing a dynamic model to simulate policy impacts including expanding long-term care insurance and popularizing early dementia screening would help policymakers evaluate cost-effectiveness [4]. The model can build on the study’s existing probit regression framework, incorporating policy implementation data from pilot provinces to quantify how interventions alter prevalence trajectories. Third, disaggregating costs by ADRD subtypes such as Alzheimer’s disease and vascular dementia and care settings including home, community, and institution would address heterogeneous care needs [5]. Subtype information can be linked to clinical diagnosis data from tertiary hospitals, while care setting distinctions can be derived from CHARLS’s assessments of activities of daily living and instrumental activities of daily living, as well as healthcare utilization records. These adjustments would strengthen the link between research evidence and equitable ADRD care implementation across China’s diverse provinces.
